# Clinical application of liquid biopsy based on circulating tumor DNA in non-small cell lung cancer

**DOI:** 10.3389/fphys.2023.1200124

**Published:** 2023-06-07

**Authors:** Liu Xin, Yang Yue, Ren Zihan, Cui Youbin, Lu Tianyu, Wang Rui

**Affiliations:** Department of Thoracic Surgery, First Hospital of Jilin University, Changchun, China

**Keywords:** liquid biopsy, non-small cell lung cancer, early diagnosis, prognosis, recurrence, adjuvant therapy

## Abstract

Lung cancer is a widely occurring and deadly malignancy, with high prevalence rates in China and across the globe. Specifically, non-small cell lung cancer (NSCLC) represents about 85% of all lung cancer cases. The 5-year disease-free survival rate after surgery for stage IB-IIIB NSCLC patients (disease-free survival, DFS) has notably declined from 73% to 13%. Early detection of abnormal cancer molecules and subsequent personalized treatment plans are the most effective ways to address this problem. Liquid biopsy, surprisingly, enables safe, accurate, non-invasive, and dynamic tracking of disease progression. Among the various modalities, circulating tumor DNA (ctDNA) is the most commonly used liquid biopsy modality. ctDNA serves as a credible “liquid biopsy” diagnostic tool that, to a certain extent, overcomes tumor heterogeneity and harbors genetic mutations in malignancies, thereby providing early information on tumor genetic alterations. Despite considerable academic interest in the clinical significance of ctDNA, consensus on its utility remains lacking. In this review, we assess the role of ctDNA testing in the diagnosis and management of NSCLC as a reference for clinical intervention in this disease. Lastly, we examine future directions to optimize ctDNA for personalized therapy.

## 1 Introduction

Currently, lung cancer continues to be the primary contributor to cancer-related mortality, with non-small cell lung cancer (NSCLC) being its most common subtype. Approximately 1,918,030 new cases of cancer and 609,360 cancer deaths occurred in the past year 2022, including an estimated 350 lung cancer deaths per day as projected by the American Cancer Society (Sung et al., 2021; Siegel et al., 2022). Furthermore, China, the most populous country globally, has witnessed an increasing trend of lung cancer-induced morbidity and mortality over recent decades (Sung et al., 2021). While imaging and tissue aspiration biopsy represent the most popular modalities used in clinical practice, their utilization for preoperative diagnosis and prognostic assessment of lung cancer is beset by numerous limitations including the need for detectable lesions, high false positives, radiation exposure, hysteresis quality, and other deficiencies (Jabbari et al., 2019; Werner et al., 2022). Therefore, the current need for early detection, early treatment, and favorable prognosis of NSCLC cannot be adequately addressed by solely relying on imaging and histology biopsies.

Recent years have witnessed the integration of fundamental sciences such as molecular biology and genomics into the study of cancer, leading to the emergence of liquid biopsy as a cutting-edge hotspot in the field of precision medicine in oncology. Notably, liquid biopsy based circulating tumor DNA (ctDNA), recommended by the College of American Pathologists (CAP), the International Association for the Study of Lung Cancer (IASLC), and the Association for Molecular Pathology (AMP) for molecular testing of NSCLC patients, supplies real-time molecular tumor trail information to guide the diagnosis and prognosis of tumors, as well as comprehensive results of genetic testing for precision cancer treatment (Wang et al., 2022a; Bosse et al., 2022; Esfahani et al., 2022). Lu et al., for example, utilized the ctDNA sequencing-based tumor mutation index (*TMI*) model to determine which NSCLC patients would benefit from receiving monotherapy with docetaxel or atezolizumab (Lu et al., 2022).

ctDNA detection techniques have played a significant role in the extensive use of ctDNA detection. Initially, ctDNA detection relied heavily on real-time Polymerase Chain Reaction (qPCR) and Next-Generation Sequencing (NGS) technologies (Bennett et al., 2022; O'Sullivan et al., 2022; Yuwono et al., 2022). However, recent technological advancements have resulted in the development of several new detection techniques that offer greater sensitivity and wider applicability for the detection of ctDNA including the use of BEAMing Technology to detect *IDH1* mutations in patients with gliomas; the use of CAPP-Seq to elucidate the genomic landscape of ctDNA in metastatic extramammary Paget’s disease; the use of Whole Exome Sequencing (WES) to study the therapeutic effect of late adjuvant setting in hormone receptor-positive breast cancer; and the development of a Droplet Digital PCR (ddPCR)-based tumor detection method (Cabezas-Camarero et al., 2022; Henriksen et al., 2022; Lipsyc-Sharf et al., 2022; Sawamura et al., 2022).

**FIGURE 1:**
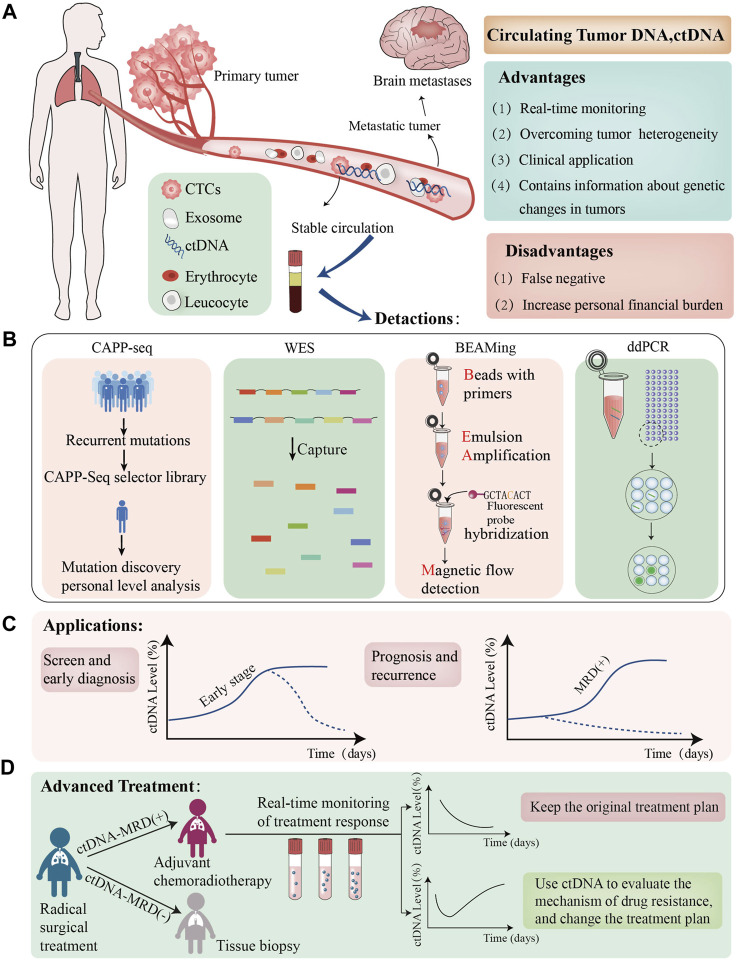
The role of ctDNA in NSCLC. **(A)**; We have presented the Advantage and disadvantages of utillizing ctDNAtechnolgy, which are dentoted by green and red boxes respectively; **(B)** we have listed the lattest ctDNA detection technolgies; **(C)**; we evaluated the clinical application of ctDNA in patients with NSCLC the graphs demonstrate varying ctDNA Kinetics, dotted black lines demostrete potential cases of early ctDNA clerance; **(D)**:ctDNA-MRD can guide adjuvant treatment in advanced non-small cell lung cancer patients, the graphs demonstreat varying ctDNA Kinetics. ctDNA+:ctDNA positive result,ctDNA-:ctDNA negative result CTCs, circulating tumour cells MRD minimalresidual CAPP-seg,cancer personalized profiling by deep sequencing,WES, Whole exome Sequencing, ddPCR, Droplet Digital PCR.

In this review, we will employ ctDNA testing as an entry point for liquid biopsy in advancing the clinical implementation of ctDNA analysis for early detection, prognostic assessment, recurrence monitoring, and postoperative adjuvant therapy in patients with NSCLC (Figure 1). When the amount of total tumor DNA in the blood reaches a certain threshold, it can be classified as ctDNA. Nevertheless, if the ctDNA concentration is unclear, blood DNA analysis should be termed as cfDNA detection. Therefore, at the end of the article, we also summarized the applications of cfDNA in tumors. Additionally, we will explore the potential future applications of ctDNA technology.

## 2 Relationship between ctDNA and liquid biopsy

A laboratory procedure known as liquid biopsy involves the collection of blood-based bodily fluids in order to obtain biomarkers that can be used for detection and analysis to gather information about cancer (Siravegna et al., 2019). Other bodily fluids contain specific neoplastic information, such as cerebrospinal fluid (CSF) for tumors of the central nervous system (Wu et al., 2022), saliva for tumors of the head and neck (Shen et al., 2020), pleural effusion for tumors of the thorax and metastases (Sorolla et al., 2021), ascites for tumors of the abdomen and metastases (Han et al., 2020), and urine for tumors of the urinary tract (Zhang et al., 2021). Circulating tumor DNA (ctDNA), circulating tumor cells (CTCs), and exosomes in peripheral blood are the primary targets of liquid biopsy. CTCs are shed into the bloodstream from either primary or metastatic tumors. CTCs have a close relationship with the development and prognosis of metastatic tumors (Chen et al., 2022), which can be detected in various cancer by a series of molecular markers represented by *EpCAM* (Zavridou et al., 2020). Furthermore, exosomes are a heterogeneous group of membrane-structured vesicles actively released by most cells. Increasing studies have shown that various disease-related proteins and nucleic acids are loaded into exosomes and differentially expressed in tumors of different origin (Hoshino et al., 2020; Preethi et al., 2022). For instance, exosomal CD63 was reported to be high in ovarian cancer while low in lung cancer (He et al., 2014). Using ctDNA testing as a starting point, this review will examine the current clinical use of liquid biopsy in NSCLC patients.

DNA fragments known as ctDNA harbor tumor-specific information such as point mutations, chromosomal rearrangements, copy number aberrations, methylation, and gene expression, and are shed by tumor cells into the peripheral circulatory system (Keller et al., 2021). The main sources of ctDNA in tumor cells include apoptosis, necrosis, active release, phagocytosis, and cell detachment (Thierry et al., 2016). Over the years, numerous studies have confirmed the application of ctDNA in tumors. Clark et al. developed a new sequencing method (FoundationACT) for detecting short variants (base substitutions, short insertions/deletions) at low allele frequencies in plasma ctDNA through next-generation sequencing. The method exhibited a >99% overall sensitivity (95% CI, 99.1%–99.4%) for short variants at low allele frequencies >0.5% (Clark et al., 2018). Notably, sequencing sensitivity of this technology remained as high as 70% even when low allele frequencies were between 0.25% and 0.5% (Clark et al., 2018). A high concordance between the results of the combined genomic profiling (CGP) of tumor tissue samples and blood samples from 96 paired specimens of colorectal cancer (CRC) patients was demonstrated (Li et al., 2019). In the plasma of several cancer patients, specific mutations including *TP53* mutations in blood samples from patients with small cell lung cancer (SCLC), *KRAS* mutations reported in tests of NSCLC patients, and *BRCA1* mutations in blood samples from patients with breast cancer were detected by Pessoa et al. (Pessoa et al., 2020). They concluded that ctDNA is a superior option compared to prior tumor biomarkers. Taieb et al. (Taieb et al., 2021) conducted a study on 1,017 randomized resectable stage III colon cancer patients and found that the 3-year DFS rates were 66.39% and 76.71% in 140 ctDNA-positive cases compared to 877 ctDNA-negative cases (*p* < 0.05), indicating that ctDNA is an independent prognostic marker. Based on the prognostic value of ctDNA in cancer patients, the clinical applicability of ctDNA monitoring can be examined further. Chen et al. (Chen et al., 2019) found that ctDNA testing on postoperative day 3 after radical resection could be used as a baseline value for postoperative surveillance of NSCLC.

## 3 ctDNA and early diagnosis

With a sensitivity of 93.7% in screening high-risk groups (55–75 years old, more than 30 packs/year smokers), low-dose CT (LDCT) has emerged as the standard test method for lung cancer screening and early detection (Aberle et al., 2011). However, research conducted by the USNLST has revealed that the LDCT screening group has a false-positive rate of 96.4% (de Koning et al., 2014), which could lead to significant levels of physical and psychological stress for patients due to the frequent reviews of LDCT results and the potential need for tissue biopsy. Hence, there is an urgent need to explore novel screening methods that could complement, or even replace, LDCT in the early detection of lung cancer. ctDNA testing, which represents a new testing tool for liquid biopsy, has the potential to detect early abnormal signals of most cancers. Notably, several studies have found that commonly used tumor biomarkers, such as carcinoembryonic antigen, alpha fetoprotein, glycoantigen 125, glycoantigen 199, and glycoantigen 72-4, exhibit lower sensitivity than ctDNA (Yang et al., 2018). Zhang et al. (Zhang et al., 2019) suggest that tumor-associated gene mutations, including *EGFR, KRAS, ALK, HER2, BRAF, ROS1, RET*, and others, could predict the emergence of primary tumors by indicating ctDNA gene changes, thus emphasizing the potential of the ctDNA testing approach.

Given that the amount of ctDNA in the early stages of NSCLC is significantly lower than in late-stage disease, there is a pressing need for new techniques and methods to enhance the sensitivity and accuracy of ctDNA-based early detection. In this regard, Liu et al. (Liu et al., 2019) developed a new method for ssDNA library preparation and hybrid-capture-based cfDNA sequencing (single-strand library preparation and hybrid-capture-based cfDNA sequencing, SLHC-seq) for cfDNA sequencing of pancreatic cancer and healthy controls in a clinical cohort. The study reported (Liu et al., 2019) that this ultra-sensitive ssDNA library preparation method is more efficient in detecting and quantifying ultra-low abundance mutations in the genome than commonly used double-stranded library preparation methods, especially in the context of low sample sizes. This new method is highly sensitive and is more accurate than traditional sequencing and PCR methods, making it an important tool for early-stage disease detection where tumor cfDNA levels are low. The success of this study is partly attributable to the removal of high-molecular-weight genomic DNA that is often produced when blood is withdrawn and centrifuged for plasma extraction. Professor Julie Earl from the Cajal Institute in Spain is optimistic about the clinical translation of this research, especially its potential positive impact on cancer diagnosis and treatment (Earl, 2019). In addition, Liu et al. employed emerging technologies to design an ultrasensitive electrochemical biosensor (AuNPs/Ni-CAT/CB/PPGE sensors) with a broad linear response range of 1 × 10^−15^ M to 1 × 10^−6^ M for ctDNA detection (Liu et al., 2022). Cao et al. also developed a highly sensitive and simultaneous detection of ctDNA related to NSCLC using a catalytic hairpin assembly strategy in a SERS microfluidic chip (Cao et al., 2022b).

In addition to low sensitivity and accuracy issues concerning early detection, false positives have also emerged as a critical problem. Recently, Chabon et al. (Chabon et al., 2020) developed a novel diagnostic model, Lung-CLiP, which could distinguish tumor-derived and clonal hematopoiesis (CH)-derived cfDNA fragments. This approach involves targeted sequencing of plasma cfDNA and matched leukocyte DNA, including the integration of single nucleotide variants (SNVs) and copy number analysis with machine learning models. The researchers observed a strong correlation between metabolic tumor volume and the sensitivity of Lung-CLiP, with sensitivities of approximately 16% (95% CI: 4%–24%), 52% (95% CI: 32%–72%), and 80% (95% CI: 60%–96%) for tumors with volumes of 1 mL, 10 mL, and >100 mL, respectively (Chabon et al., 2020). A significant highlight of this study is the improvement of the cfDNA extraction and sequencing method, CAPP-seq. Results from lymphocyte sequencing were used to correct for acquired mutations in cfDNA with error correction before sequencing. These findings indicate that simultaneous detection of plasma ctDNA and leukocyte DNA is important to exclude the false negative ctDNA detection caused by CH.

Methylation-based ctDNA analysis presents an innovative approach that addresses the current challenge of early detection of ctDNA (Chen C. et al., 2020; Chen et al., 2020b). The Circulating Cell-free Genome Atlas (CCGA; NCT02889978) study, developed by the GRAIL team, has confirmed that targeted methylation-based ctDNA detection plays a crucial role in multi-cancer early detection. In the third CCGA substudy, Klein et al. outlined in-depth experimental data for MCED concerning breast, colorectal, cervical, lung, and prostate (Klein et al., 2021). The specificity for cancer signal detection was found to be 99.5% [95% confidence interval (CI): 99.0%–99.8%]. The overall sensitivity for cancer signal detection was 51.5% (49.6%–53.3%) and increased with the stage of cancer. Stage I-III sensitivity was 67.6% (64.4%–70.6%) for the pre-specified cancers that account for approximately two-thirds of annual United States cancer deaths. In a recently published paper (Jamshidi et al., 2022) the GRAIL study confirmed the precision of MCED using the methylation clinical LOD (limit of detection) which was 1.33 × 10^−4^ cTAF (targeted circulating tumor fraction) at 98% specificity. Jamshidi et al. (Jamshidi et al., 2022) have explained that methylation is the optimal choice because it is a widespread signal across the genome. The methylation patterns along each fragment contain a robust tumor-specific signal, which can be easily detected above normal genomic background variation. Thus, it may enable the methylation signal to be detected at lower cTAF levels than other features of the cancer genome.

The use of stacked ensemble approaches to integrate genomic features of cfDNA from whole-genome sequencing (WGS) has shown promising initial results in detecting early colorectal adenocarcinoma (Ma et al., 2021). However, is this approach appropriate for lung cancer screening? Lin Xu et al. from Jiangsu Cancer Hospital built a stacked ensemble machine learning model integrating omics features of five cfDNA fragments (Wang et al., 2022c). Xu et al. extracted five different fragment features from WGS data: copy number variation (CNV), fragment size coverage (FSC), fragment size distribution (FSD), end sequence (EDM), and break point sequence (BPM). They then used each cfDNA fragment feature to build its base model, applying five base algorithms, including Generalized linear model (GLM), gradient boosting machine (GBM), random forest, deep learning, and XGBoost. Finally, they built a stacked ensemble model by integrating the omics features of plasma cfDNA fragments and five machine learning algorithms. This model distinguished between cancer and non-cancer samples with both high sensitivity and specificity of 92.5% and 94.2%, respectively. Remarkably, this model’s prediction performance was excellent for early-stage lung cancer, with a sensitivity of 83.2% for stage I lung cancer and a sensitivity of 85% for tumors smaller than 1 cm (Wang et al., 2022c). Therefore, this study established a stacked ensemble model based on cfDNA fragments with high sensitivity, stability, and robustness, which is useful for the early detection of NSCLC. In addition, some scholars have suggested combining ctDNA methylation with fragmentation to provide a more clinical basis for lung cancer’s early detection (Kim Y. J. et al., 2022; Nguyen et al., 2022).

The idea of a “drop blood test for cancer” once sounded like a fairytale. However, recent research by Efrat Shema and her colleagues in Israel has given us hope (Fedyuk et al., 2023). The researchers developed a single molecule-based liquid biopsy method called the plasma fractionation micronucleus epigenetics technique (EPINUC) to analyze the data from multiple epigenetic parameters of plasma samples (<1 mL) (Fedyuk et al., 2023). This approach allowed for high-resolution detection of six active and repressive histone modifications and their ratios and combination patterns on millions of individual nucleosomes through single-molecule imaging. By doing so, the researchers broke traditional technical limitations and gained insights from a limited amount of (<1 mL) liquid biopsy material, enabling effective acquisition of clinically relevant multi-layer information. Finally, the researchers combined the molecular biology of cancer with artificial intelligence algorithms to effectively distinguish between those who had cancer and those who did not, achieving a 98% accuracy rate (Fedyuk et al., 2023). To enhance the clinical utility of this study, further research may be needed to expand the types of cancer included.

In conclusion, the remarkable contribution of ctDNA to the early detection of NSCLC, such as through gene mutation, methylation, fragmentation, and nucleosome surface epigenetic characteristics, suggests that ctDNA detection is a viable option when traditional detection methods are not feasible for the early detection of lung cancer. While ctDNA is a promising tool for early detection, technical challenges must be overcome. Unlike molecular residual disease (MRD) detection, ctDNA lacks prior knowledge of tumor tissue and specific mutations, and the commonly used technique WGBS can result in extensive DNA damage, strand breaks, and loss of the DNA template, requiring high input and reducing effective data (Beck et al., 2022). Additionally, delays in plasma preparation, storage time, methods, and conditions may interfere with ctDNA fragmentation patterns and increase the risk of leukocyte genomic DNA contamination (Song et al., 2022).

## 4 ctDNA and the prognosis of NSCLC

At present, curative surgical resection, specifically “lobectomy plus systematic mediastinal lymph node dissection,” remains the optimal treatment choice for patients with stage I, II, and some stage IIIa NSCLC. However, despite such treatment, approximately 30%–55% of patients experience treatment failure due to recurrence or metastasis revealing the presence of molecular level MRD that cannot be detected by traditional imaging methods (Goldstraw et al., 2016). The definition of MRD is clearly explained in the 18th lung cancer summit’s expert consensus on lung cancer MRD formed in 2021. MRD refers to abnormal tumor-originating molecules that cannot be detected using traditional imaging or laboratory methods but can be identified via liquid biopsy. It is indicative of the continued existence of lung cancer and potential clinical progression. Currently, numerous studies have proven the application of circulating tumor DNA-minimal residual disease (ctDNA-MRD) during the perioperative period of lung cancer patients (Cai et al., 2021; Peng et al., 2021; Pellini and Chaudhuri, 2022). For example, regarding prognostic analysis, MRD detection demonstrated a high predictive capacity for disease relapse.; with respect to treatment response evaluation, a robust relationship was identified between clearance of ctDNA and pathological response, both of which were correlated with longer survival following neoadjuvant therapy (Shen et al., 2022). Gao et al. proposed an innovative dual-signal amplification approach that centers on a pump-free Surface-enhanced Raman scattering microfluidic chip, and utilizes catalytic hairpin assembly technology, to enable real-time tracking of the variations in ctDNA-MRD (Cao et al., 2022a). We used a chart to record the clinical value of MRD in postoperative NSCLC patients over the past 3 years (Table 1).

**TABLE 1:** The application of MRD detection in postoperative patients with NSCLC.

Author (year)	Title	Study type	Number of included samples	Definition of postoperative MRD positivity	Postoperative MRD positive rate	Predicting relapse in advance with post-operative MRD	The ratio of post-operative MRD positive vs negative patients experiencing relapse
Li et al. (2022)	Perioperative circulating tumor DNA as a potential prognostic marker for operable stage I to IIIA non-small cell lung cancer	prospective	123 patients with resectable stage I to IIIA NSCLC	1 month after surgery	10.3%	8.71 months	Significant reduction in RFS(HR:3.46; 95% CI:1.59–7.55; *p* < 0.05)
Gale et al. (2022)	Residual ctDNA after treatment predicts early relapse in patients with early-stage non-small cell lung cancer	prospective	88 patients with early-stage NSCLC (48.9%/28.4%/22.7% at stage I/II/III)	Week 2	17.0%	212.5 days	64.3% vs. 15.7%
							*p* < 0.05
Wang et al. (2022a)	Circulating tumor DNA integrating tissue clonality detects minimal residual disease in resectable non-small-cell lung cancer	prospective	128 patients with stage I-III NSCLC	Week 1	32.3%	145 days	73.5% vs. 16.5%
							*p* < 0.05
Qiu et al. (2021)	Dynamic recurrence risk and adjuvant chemotherapy benefit prediction by ctDNA in resected NSCLC	prospective	116 patients with operable NSCLC	1 month after surgery and before the start of ACT	21.2%	88 days	Significant reduction in RFS (HR:4.0; 95%CI:2.0–8.0; *p* < 0.05)
Xia et al. (2022)	Perioperative ctDNA-Based molecular residual Disease detection for non-small cell lung cancer: a prospective multicenter cohort study (LUNGCA-1)	prospective	330 patients with stage I-III NSCLC	3 days and/or 1 month postoperatively	8.81%	MRD positivity was a strong predictor of recurrence	80.8% vs. 16.2%; *p* < 0.05
Zhang et al. (2022)	Longitudinal undetectable molecular residual disease defines potentially cured population in localized non-small cell lung cancer	prospective	216 patients with operable NSCLC	1 month after surgery	652 cases were postoperative	3.4months	PPV:89.1%
							NPV:96.8%

NSCLC, non-small-cell lung cancer; MRD, minimal residual disease; ACT, adjuvant therapy; PFS, progression free survival; HR, hazard ratio; 95% CI, 95% confidence interval; PPV, positive predictive value; NPV, negative predictive value.

Li and colleagues conducted a prospective clinical study in perioperative NSCLC patients, which revealed that ctDNA-MRD (+) patients detected within the first month after surgery were associated with a shorter RFS. Additionally, continuous monitoring of ctDNA enabled the detection of NSCLC recurrence approximately 8.71 months earlier than traditional radiological detection (Li et al., 2022). In a retrospective study, Gale et al. (Gale et al., 2022) found that ctDNA detection had a clinical specificity of over 98.5% and facilitated the early detection of recurrence prior to clinical detection of the primary tumor by a median of 212.5 days. In the study by Wang and colleagues (Wang et al., 2022b) over 1,000 tumor tissue and plasma samples were analyzed, with MRD sampling and testing performed before surgery, 7 days after surgery, and every 3 months during the dynamic monitoring stage. The results indicated that ctDNA detection could detect MRD positivity an average of 145 days earlier than imaging for recurrence detection. Therefore, one of the critical directions in MRD research would be to develop guidance for adjuvant treatment decisions based on MRD status. This approach would enable clinicians to select and enrich subpopulations that could genuinely benefit from adjuvant treatment. By doing so, it could potentially reduce the population of cancer patients who go on to experience postoperative recurrence, ultimately leading to better clinical outcomes.

The CALIBRATE-NSCLL study utilized the TAG-seq detection technology on 116 operable NSCLC patients, of whom 66 received adjuvant therapy as per guidelines. Preoperative blood and surgical specimens were collected as baseline samples, and peripheral blood samples were collected within 1 month after surgery, after adjuvant chemotherapy, and every 3 months thereafter for dynamic monitoring (Qiu et al., 2021). Qiu and colleagues made significant findings in this prospective study (Qiu et al., 2021). Firstly, ctDNA-MRD (+) within 1 month after surgery was an independent predictor for PFS post-surgery. Secondly, among MRD positive patients after surgery, the risk of relapse was significantly lower in patients who received adjuvant therapy than in those who did not. Thirdly, MRD negative patients after surgery did not derive significant clinical benefits either with or without adjuvant therapy (ACT). Thus, the study indicates that patients who are MRD positive after surgery may benefit from ACT, while those who are MRD negative do not reap any significant benefits from this intervention. Fourthly, the risk of relapse in MRD positive patients after adjuvant therapy is significantly higher than that in MRD negative patients after adjuvant therapy, indicating that MRD status after adjuvant therapy can predict the risk of relapse and facilitate clinical intervention after ACT. Finally, dynamic ctDNA changes can predict relapse with 88 days of precision earlier than imaging. Overall, the study demonstrates that ctDNA-MRD can serve as a dependable biomarker for risk stratification and early detection of relapse after surgery and ACT treatment for NSCLC. Furthermore, the study data further suggests that postoperative ctDNA-MRD analysis can guide ACT treatment decisions and avoid overtreatment of patients who are unlikely to benefit from ACT.

In the prospective multicenter cohort study conducted by Xia et al., dynamic ctDNA detection was performed in lung cancer surgery patients (LUNGCA-1) (Xia et al., 2022). The study recruited 330 NSCLC patients at I-III stages and collected 950 plasma samples at three perioperative time points: preoperative, postoperative day 3 (ranging from 2 to 15 days), and 1 month after surgery (ranging from 3 to 6 weeks). A 769-gene panel was used for second-generation sequencing of somatic mutations in tumor tissues and plasma samples for ctDNA-MRD analysis. The study defined “MRD positive” as the detection of ctDNA at postoperative day 3 and/or 1 month. MRD-positive patients had a higher recurrence rate than MRD-negative patients (80.8% vs 16.2%, *p* < 0.001). After multivariate Cox proportional hazards regression analysis, ctDNA-MRD had a higher relative contribution to the prediction of RFS compared to all clinical-pathological variables including TNM stage, tumor size, and histology (Xia et al., 2022). Finally, the researchers concluded through the comparison of 17 patients receiving adjuvant therapy that MRD-positive patients, rather than MRD-negative patients, benefited from postoperative adjuvant therapy (Xia et al., 2022). Due to current controversies on the use of adjuvant therapy for stage IB NSCLC, ctDNA-MRD detection in this study provides a useful reference for whether this group of patients needs to receive postoperative adjuvant therapy as 67% of operable NSCLC patients were at the I stage.

Recently, Professor Wu and colleagues conducted a prospective, non-interventional, observational study to explore the role of MRD monitoring in I-IIIA stage NSCLC patients after radical surgery. The study analyzed preoperative blood samples from 261 patients with stage I-III NSCLC and successfully analyzed 256 tumor tissue and 652 postoperative blood samples. During the postoperative monitoring period, 224 patients (91.4%) did not detect MRD, most of whom (n = 194) remained disease-free, with a negative predictive value (NPV) of 86.6%. Upon integrating longitudinal time points, the NPV increased further to 96.8%. Thus, 96.8% of the patients who did not detect longitudinal MRD remained disease-free at the last follow-up visit, thereby defined as “potential cure population.” (Zhang et al., 2022). In analyzing 85 high-risk stage II-III patients, the hazard rate curve for detectable MRD or disease recurrence reached a peak at the 18th month (Zhang et al., 2022). Therefore, patients remaining undetectable of MRD for over 18 months represent a population of possible cure. In addition, the study also analyzed 55 patients receiving adjuvant therapy. Compared with 10 MRD positive patients, the researchers concluded that MRD negative patients may not need adjuvant therapy (Zhang et al., 2022). Moreover, they found that 5 of the 10 MRD positive patients did not achieve ctDNA clearance during adjuvant treatment (unclearing group), 2 experienced temporary ctDNA clearance, but then tested positive for ctDNA (clearance elevation group), and 3 remained ctDNA negative (clearance group). Except for the clearance group, there seemed to be no difference in DFS between the other two groups (Zhang et al., 2022). Therefore, we should not evaluate the efficacy of adjuvant therapy based on the clearance rate of ctDNA at a single time point.

The latest study published in Nature shows that postoperative ctDNA serves as a marker for the developing systemic nature of disease recurrence and metastasis (Abbosh et al., 2023). This research collected 1,069 plasma samples from 197 patients and analyzed blood samples collected within 120 days after surgery. It was found (Abbosh et al., 2023) that 25% of patients had ctDNA detected in their plasma, including 49% of all patients who experienced clinical recurrence. In the remaining 20% of milestone (120-day postoperative) negative patients, ctDNA monitoring over 3–6 months revealed an impending disease recurrence. The researchers also found that (Abbosh et al., 2023) detecting ctDNA in preoperative blood samples can distinguish biologically less aggressive and clinically favorable lung adenocarcinomas. To analyze ctDNA detection results, the researchers developed a bioinformatics tool called ECLIPSE, which is used for noninvasive tracking of tumor clone features. ECLIPSE can identify patients with multi-clone metastasis, which is linked to poor clinical outcomes (Abbosh et al., 2023). The collection of preoperative plasma can differentiate metastatic and non-metastatic clones, providing new insights into using low-level ctDNA analysis in plasma for evaluating cancer metastasis.

In fact, a large number of current prospective studies are non-interventional, and We also need more interventional clinical studies to prove that ctDNA-MRD biomarkers can guide patients to avoid adjuvant chemotherapy through several peripheral blood extractions according to ctDNA test results, which can save nearly half of the patients from chemotherapy while still receiving survival benefits. Recently, a prospective, interventional study on stage II colon cancer has guiding significance for postoperative MRD intervention research in NSCLC patients. The study proved that the adjuvant therapy strategy guided by MRD can reduce the number of patients receiving adjuvant chemotherapy without affecting RFS (Tie et al., 2022). Additionally, we anticipate the final results of the MOTION-NSCLC large-scale prospective, multi-center interventional MRD study, led by Professor Wu Yilong of the First Affiliated Hospital of Guangdong Province.

## 5 ctDNA and the adjuvant therapy of NSCLC

The concept of cancer precision medicine aims to develop therapies that target only cancer cells without harming normal tissues, based on the molecular and immune characteristics of the tumor at the corresponding disease stage. Two approaches, gene-targeted therapy and immunotherapy, are currently being utilized to achieve this goal. The utilization of ctDNA-based liquid biopsies has facilitated exploration into personalized biomarkers that predict durable clinical benefits with treatment, leading to research in longitudinal monitoring of clinical outcomes (Beagan et al., 2020; Mitsudomi et al., 2022; Reichert et al., 2023). For example, in ctDNA and tissue samples from 33 patients with ctDNA in their blood, genomic alterations were detected in both tissue and ctDNA in 64% of cases, including 78% of short variants and all rearrangements, which suggest that ctDNA testing may be particularly useful in detecting progression during targeted therapy (Schrock et al., 2019).

Provencio et al. conducted a large retrospective study using ctDNA dynamics to evaluate the efficacy of first-line osimertinib compared to first- or second-generation tyrosine kinase inhibitor (TKIs) resistance followed by osimertinib treatment (Provencio et al., 2021). During the study period, the median OS of 129 patients treated with non-osimertinib as second-line therapy was 22.8 months (95%CI: 16.1-NR) after first-line treatment with first-generation or second-generation TKI. Sixty patients were treated with gefitinib, erlotinib or afatinib as first-line treatment, and the median OS of patients treated with osimertinib as second-line treatment was 32 months (95%CI: 23.9-NR). The median OS of 39 patients treated with osimertinib as first-line treatment was not reached (95%CI: 17.0-NR), with statistically significant difference (*p* < 0.05). These results provide evidence of the superiority of osimertinib in patients with EGFR-mutated advanced NSCLC (Provencio et al., 2021). In addition, the study utilized ctDNA to identify patients who may benefit from sequential *EGFR*-TKI therapy, defined as MAF (mutant allele frequency) < 7% at baseline with better RFS (HR = 0.51; 95%CI: 0.34–0.78) and OS (HR = 0.38; 95%CI: 0.23–0.64). Consequently, evaluating pretreatment levels of ctDNA could aid in identifying patients with low risk who may benefit from *EGFR*-TKI therapy. We determined that a robust treatment response was characterized by a minor allele frequency (MAF) of <7% at the time of diagnosis and a negative ctDNA result at either the third or the sixth month post-treatment. Patients with a high response had better progression-free survival (HR = 0.30; 95%CI: 0.19–0.49) and OS (HR = 0.22; 95%CI: 0.12–0.42). Lastly, the study demonstrated that ctDNA clearance along with low baseline ctDNA levels identifies the group of patients with the best survival (Provencio et al., 2021).

Nakamura et al. quantified ctDNA to monitor the development of resistance during treatment with the targeted drug afatinib and found that: *EGFR* amplification was relatively resistant to afatinib at concentrations below 0.5 nM. The study concludes that the absence of *EGFR* copy number gain detected in ctDNA can serve as a predictive marker of long-term response to afatinib (Nakamura et al., 2022). A *post hoc* analysis of a pivotal phase 2 study (NCT02897479) monitored ctDNA biomarkers in savolitinib treated patients with advanced NSCLC harbouring *MET* exon 14 skipping alterations (Yu et al., 2022). Patients with detectable baseline *METex14* exhibited worse PFS (HR = 1.77; 95%CI = 0.88–3.57; *p* = 0.108) and OS (HR = 3.26; 95% CI = 1.35–7.89; *p* = 0.006) than those without (Yu et al., 2022). Among 24 patients with baseline detectable *METex14* and evaluable postbaseline samples, 13 achieved *METex14* clearance post-treatment. Median time to first clearance was 1.3 months (range, 0.7–1.5). *METex14* post-treatment clearance was associated with better ORR (92.3%; 95%CI = 64.0–99.8 *versus* 36.4%; 95%CI = 10.9–69.2; *p* = 0.0078), PFS (HR = 0.44; 95%CI = 0.2–1.3; *p* = 0.1225) and OS (HR = 0.31; 95%CI = 0.1–1.0; *p* = 0.0397) *versus* non-clearance. Among 22 patients with disease progression, 10 acquired pathway alterations (e.g., in *RAS/RAF* and *PI3K/PTEN*) alone or with secondary *MET* mutations (*D1228H/N* and *Y1230C/H/S*). Therefore, we can conclude that ctDNA biomarkers may allow for longitudinal monitoring of clinical outcomes with savolitinib in patients with *METex14*-positive. Notably, a baseline *METex14* that is undetectable or clearance post-treatment may indicate promising clinical outcomes, whereas resistance to savolitinib may be explained by secondary *MET* mutations and other acquired gene modifications (Yu et al., 2022).

A prospective, multicenter, open-label, single-arm, phase 2 nonrandomized clinical trial (NCT03346811) has solved the problem that a certain proportion of patients with clinically diagnosed advanced lung cancer cannot obtain pathological diagnosis, which solves the clinical problem of precision treatment (Xu et al., 2022). Of the 116 patients enrolled, patients received oral Icotinib (125 mg three times daily) until disease progression, death, or discontinuation for various reasons (e.g., toxic effects and withdrawal of consent). The primary endpoint was objective response rate (ORR). The secondary endpoints included progression-free survival (PFS), overall survival (OS), and disease control rate (DCR). The median follow-up time was 36.3 (30.2–40.7) months. The ORR was 52.6% (95%CI = 43.1%–61.9%). The median PFS and OS were 10.3 months (95%CI = 8.3–12.2) and 23.2 months (95%CI = 17.7–28.0), respectively (Xu et al., 2022). The DCR was 84.5% (95% CI, 76.6%–90.5%). In conclusion, this study demonstrated that using ctDNA-based *EGFR* genotyping can be used as an effective decision-making tool for selected clinical situations in patients with clinically diagnosed advanced lung cancer of unknown pathological status.

In fact, the successful application of gene targeted therapy is exemplified by its success in treating Chronic Myeloid Leukemia (CML). CML treatment involves three critical elements: the discovery of the underlying molecular genetic defect (*BCR-ABL* fusion gene); development of therapies to attenuate the enhanced kinase activity caused by this defect (imatinib and second/third generation *BCR-ABL* kinase inhibitors); and the use of imatinib in newly diagnosed diseases that have not undergone genomic evolution. Currently, the success of targeted therapies for cancer is similar to the first two components of therapy in CML. However, the impact on outcomes may be limited, particularly as these targeted therapies are frequently employed in metastatic disease, in which multiple adjuvant drivers of malignancy have already been established, rather than in newly diagnosed cases. In this context, the rise of preoperative neoadjuvant therapy in clinical practice holds promise (Janjigian et al., 2021).

Currently, numerous studies have provided evidence that ctDNA-based biomarkers are essential for guiding immunotherapy and predicting its subsequent efficacy (Wang et al., 2022c; Peters et al., 2022; Sun et al., 2022). One such study by Han et al. employed ctDNA to forecast the effectiveness of sintilimab plus docetaxel in the second-line treatment of advanced NSCLC (Han et al., 2022). Blood samples were prospectively collected at baseline and after 2 cycles of treatment (6 weeks after treatment) to describe the landscape of high-frequency genomic profiles at baseline and week 6 (Han et al., 2022). Major molecular features of preselected genes associated with response to second-line chemoimmunotherapy were analyzed. Results indicated that patients exhibiting ctDNA clearance at the sixth week demonstrated decreased tumor volume, while those with ctDNA positive at the sixth week recorded an increase in tumor volume. Notably, patients with a positive sixth-week ctDNA had significantly shorter PFS (91 vs. NR days; *p* < 0.0001) and OS (47 vs. 467 days; *p* = 0.0039) compared to those with ctDNA clearance at week 6, leading to the conclusion that ctDNA clearance at the sixth week independently served as a risk factor for progression or death (HR = 100 (95%CI = 4.10–2503.00), *p* = 0.005) (Han et al., 2022). These findings indicate that ctDNA status and ctDNA mutation clearance could potentially serve as predictive biomarkers for sintilimab combined with docetaxel chemotherapy in pretreated advanced NSCLC patients.

Due to a significant proportion of patients being unable to provide sufficient tissue for Next-Generation Sequencing (NGS) calculations of Tumor Mutation Burden (TMB), the calculation of blood tumor mutation Burden (bTMB) from ctDNA has emerged as a more promising method (Wang et al., 2019; Chen et al., 2020c). It is a non-invasive and convenient approach that can correct for sampling bias caused by tumor heterogeneity or low tumor content, and enables dynamic monitoring of early treatment response. bTMB can screen patients with NSCLC expressing low levels of PD-L1 but are likely to benefit from immunotherapy. The B-F1RST study (NCT02848651) is the first prospective clinical study assessing bTMB as a predictive biomarker for treatment efficacy in locally advanced or metastatic NSCLC patients undergoing atezolizumab treatment (Kim et al., 2022a). The study involved 152 patients with stage IIIB-IVB NSCLC who had not received immunotherapy. Of these, 119 patients (78%) had sufficient ctDNA to produce valid bTMB results. Among them, 28 patients (24%) had bTMB scores ≥16, while 91 patients (76%) had scores <16. Results showed that patients with bTMB scores ≥16 had a median PFS of 5 months compared to 3.5 months for those with scores <16. Similarly, patients with bTMB scores ≥16 had a median OS of 23.9 months compared to 13.4 months for those with scores <16. These findings suggest that bTMB may serve as a predictive biomarker for atezolizumab therapy (Kim et al., 2022a). Furthermore, the researchers found that as the bTMB cutoff value increased, i. e., when bTMB ≥18 and bTMB ≥20, correlates with improved PFS and OS (Kim et al., 2022a). However, larger-scale research is essential to verify if higher bTMB cutoff values can enhance patient outcomes as the number of patients decreased with increasing bTMB cutoff values.

Of note, raising the bTMB threshold could benefit patients from immune therapy. The study (Si et al., 2021) firstly confirmed the practical advantages of measuring tumor mutational burden (TMB) in blood using ctDNA compared to tissue (tTMB) measurement. Importantly, the study concluded that a bTMB ≥20 mut/Mb is the optimal cutoff for clinical benefit in patients receiving first-line durvalumab (anti-PD-L1 antibody) ± tremelimumab (anticytotoxic T-lymphocyte-associated antigen-4 antibody) (Si et al., 2021).

In addition to predicting whether NSCLC patients can benefit from immunotherapy combined with chemotherapy based on the threshold of bTMB, Jiang et al. demonstrated for the first time that dynamic ∆bTMB (∆bTMB = treatment period bTMB - pre-treatment bTMB) can supplement and provide non-repetitive predictive effects to bTMB prediction results (Jiang et al., 2022). The CameL-sq study (NCT03668496) was a randomized, double-blind, multicenter phase III clinical trial that recruited 389 patients with pathologically confirmed stage IIIB-IV lung squamous cell carcinoma. Patients with bTMB values ≥75% were defined as high bTMB group, and those with bTMB values < 75% were defined as low bTMB group. The study not only demonstrated that patients with low bTMB during treatment can benefit from Camrelizumab combined with chemotherapy, thereby improving PFS and OS, but also showed how combined ∆bTMB and treatment-period bTMB can benefit patients from treatment (Jiang et al., 2022). Specifically, patients with low treatment period bTMB and ∆bTMB <0 have the longest PFS and OS, patients with low treatment period bTMB and ∆bTMB <0 or ∆bTMB ≥0 have intermediate PFS and OS, while patients with high treatment period bTMB and ∆bTMB ≥0 have the worst PFS and OS. In addition, the researchers also demonstrated how to identify patients who can benefit from long-term immunotherapy by using treatment period bTMB in patients who initially received SD (stable disease) from radiotherapy (Jiang et al., 2022). An analysis of 48 initial radiotherapy SD patients in the group treated with Caririzumab combined with chemotherapy revealed that patients with high bTMB during treatment had significantly lower PFS (median, 4.1 vs. 5.6 months; HR = 2.861, *p* < 0.05) and OS (median, 7.8 vs. 18.2 months; HR = 3.546, *p* < 0.05) compared to patients with low bTMB. For the 20 patients with initial radiotherapy SD, but with the best response of partial remission (PR), their bTMB during treatment was significantly lower than their corresponding baseline bTMB (*p* < 0.001), and the proportion of patients with treatment bTMB ≥75% was also lower than that of the SD group with the best response (10.0% vs. 32.1%). Therefore, we can conclude that patients who achieved SD from initial radiotherapy and partial remission (PR) from subsequent immunotherapy combined with chemotherapy can benefit from the treatment process of camrelizumab combined with chemotherapy compared with patients who achieved SD from initial radiotherapy and SD from subsequent treatment. In fact, this study highlights an important principle of precision medicine, which is to select the right time and circumstances for patients to benefit from immunotherapy based on ctDNA changes.

In short, the essence of immunotherapy based on ctDNA is to target the tumor cell antigens with high specificity. For example, in treating patients with high tumor mutation burden (TMB-H), it is generally believed that the new antigens generated by a large number of gene mutations, including tumor mutation antigens (TMB), neoantigens (NNA), and tumor cell surface markers, can effectively guide the patient’s immune system to attack malignant tumor cells, achieving the goal of controlling and treating NSCLC. However, not all TMB-H patients are like this, there are inevitably other factors that make immunotherapy not always successful (Wahida et al., 2023). At the same time, experts have also proposed suggestions to use immunotherapy as early as possible and to try targeted immunotherapy in combination (Wahida et al., 2023).

## 6 The clinical application of cfDNA

In fact, ctDNA is part of cfDNA, which is a mixture of nucleic acids released into the bloodstream through apoptosis, necrosis, and active secretion. cfDNA is typically found as double-stranded fragments of approximately 150–200 base pairs in length (Warton et al., 2014). Hematopoietic cells are usually the primary source of cfDNA and contribute somatic variants to the cfDNA pool. The concentration of cfDNA in healthy adults is generally very low, often less than 10 ng per ml of plasma (Warton et al., 2014). However, in cancer patients, the cfDNA concentration can vary from normal levels to many times higher (Zheng et al., 2021). To differentiate between mutations related to clonal hematopoiesis (CH) and those related to tumor-derived DNA, Chabon et al. (Chabon et al., 2020) emphasize the importance of sequencing matched leukocyte DNA and cfDNA to equivalent depths; then, they found a significantly positive correlation between the number of white blood cell (WBC) positive mutations and age, whereas there was no significant correlation between the number of WBC negative mutations and age.

Johns Hopkins University developed a liquid biopsy technology named CancerSEEK, based on cfDNA, which allows for early detection of eight common cancers, including esophageal, lung, and gastric cancers, with a median sensitivity of 70% and specificity of over 99% (Cohen et al., 2018). Zhang et al. have revealed that (An et al., 2023) DNA methylation is a crucial regulatory element of circulating cfDNA. Based on this principle, a pan-cancer diagnostic marker named the “E-index” was developed, which is based on the end distribution of cfDNA. The E-index model can effectively distinguish lung cancer samples from the control group with an AUC of 0.91. Furthermore, the E-index of metastatic lung cancer patients was significantly lower than that of non-metastatic patients, suggesting its ability to distinguish between these two groups. Furthermore, the application of nuclease-associated cfDNA markers enables the inference of tissue-of-origin and gene expression of cfDNA by combining information on cfDNA fragment coverage and fragment ends at various open chromatin regions (Chan et al., 2016; Snyder et al., 2016; Sun et al., 2019). However, while certain nucleases have been found to influence the application of cfDNA in cancer, many other nucleases, such as endonuclease G, and topoisomerase II, require further research to gain a more concrete understanding of their specific mechanisms.

Dziadziuszko et al. demonstrated the significant role of cfDNA concentration in the prognosis of advanced ALK-positive NSCLC patients (Dziadziuszko et al., 2022). Specifically, when the median plasma cfDNA concentration was >11.53 ng/mL, patients were more likely to experience disease progression and had lower overall survival rates (Dziadziuszko et al., 2022). The cfDNA in plasma binds to nucleosomes that are post-translationally modified, representing the epigenetic profile of the originating cells. The cell-free chromatin immunoprecipitation (cfChIP) technique enables the isolation and analysis of chromatin-protein interactions. By identifying tumor-specific highly expressed genes and quantifying transcript levels associated with nucleosomes, cfChIP extends the application of cfDNA in NSCLC to assess treatment resistance, cancer subtypes, and disease progression (Trier Maansson et al., 2023). For instance, H3K36me3 cfChIP can reproduce the anticipated upregulation of KRT6 in lung squamous cell carcinoma relative to adenocarcinoma, as exemplified in the blood plasma of 14 patients with lung cancer (Vad-Nielsen et al., 2020).

## 7 Discussion

ctDNA is a reliable tumor marker that plays an important role in the diagnosis and treatment of NSCLC. ctDNA can be used for diagnosis, disease monitoring, treatment strategy selection, and prognostic evaluation (Bracht et al., 2018; Roosan et al., 2021). The use of ctDNA for NSCLC screening, early diagnosis, and disease monitoring can improve early diagnosis rates and treatment efficacy assessment, while also reducing unnecessary radiation exposure and pathological diagnosis. Additionally, ctDNA can identify the true beneficiaries of adjunctive therapy, define the “potential curative population,” guide the development and adjustment of individualized treatment plans, and evaluate patient prognosis after treatment. In summary, the application of ctDNA in NSCLC has significant clinical value. However, in the clinical application of ctDNA testing, we must consider the low sensitivity of gene fusion and copy number variation, as well as the occurrence of “false-positive” results caused by CHIP mutations. Therefore, experts from the EMSO suggest that in the future, clinical gene typing tests can be used to evaluate tumor purity to enable reliable prediction of undetected results and reliable prediction of true negatives, which can be achieved through bioinformatics analysis (Pascual et al., 2022).

Currently, the final determination of ctDNA is influenced by a variety of factors, such as tumor volume, location, anti-tumor treatments (e.g., surgery, chemotherapy, radiotherapy), liver and kidney clearance rate, and contamination from hematopoietic cell-derived cfDNA during the testing process (Fernandes et al., 2021; Stejskal et al., 2023; van 't Erve et al., 2023). In 2022, the European Society for Medical Oncology (ESMO) published standardized procedures for ctDNA detection (Pascual et al., 2022). For example, during analysis, false negatives should be carefully monitored, as they may be caused by low plasma DNA levels, insufficient detection sensitivity, or “non-shedding” tumors. To better apply ctDNA testing in clinical practice, firstly, more accurate and sensitive detection techniques would be required. The researchers (Mack et al., 2020) evaluated a digital next-generation sequencing platform for detecting ctDNA in NSCLC patients, which suggests that ctDNA can increased the identification of driver mutations by 65% over standard-of-care, tissue-based testing at diagnosis. Certainly, it is necessary to evaluate the current sequencing methods. Gong et al. have comprehensively evaluated the performance of five ctDNA detection methods used for clinical and scientific research (with Integrated DNA Technologies and Burning Rock Dx sequencing demonstrating the best performance), which provides more feasibility reference for the clinical application of ctDNA (Gong et al., 2022).

Liquid biopsy, especially ctDNA detection, is increasingly being used in clinical practice, backed by sufficient evidence demonstrating its clinical utility for guiding clinical treatment of late-stage cancer gene profiles, particularly in cases where tissue biopsies are suboptimal or time-sensitive (Park et al., 2019; Wislez et al., 2021; Provencio et al., 2022). In the future, we believe that three key issues need to be addressed in order to fully leverage the potential of liquid biopsy based on ctDNA in clinical practice: 1) as genomic sequencing research continues to provide more information, clinicians also need to pay attention to the application of ctDNA in tumors. This requires a thorough understanding of patients’ genomic information to ensure that results are correctly interpreted and appropriate decisions are made, especially for difficult cases. For example, patients can benefit from information on which tumor genes they possess, the cancer patterns in their race and region, the impact of direct causes of their cancer, or the impact of dietary factors on the genome; 2) while early detection of cancer through liquid biopsy is exciting, challenges still exist. First, we must demonstrate the benefits of early detection of tumors in significantly reducing mortality, which may require large-scale prospective, intervention clinical research. Second, if liquid biopsy tests are positive early, what monitoring or follow-up guidelines should be followed; 3) although tumor-targeted therapy has brought hope for precise individualized treatment, we must understand that once a clinical target is eliminated, competition between clones for oxygen or nutrition may be reduced, which may accelerate the growth of non-targeted clones. So how to eliminate this interclonal competitive advantage and inhibit the growth of the entire tumor? In any case, ctDNA liquid biopsy-based precision individualized treatment for tumors has achieved surprising results, and we look forward to more practical clinical research to further confirm the accuracy and effectiveness of ctDNA detection.
